# A Novel Tri-Axial MEMS Gyroscope Calibration Method over a Full Temperature Range

**DOI:** 10.3390/s18093004

**Published:** 2018-09-07

**Authors:** Haotian Yang, Bin Zhou, Lixin Wang, Haifeng Xing, Rong Zhang

**Affiliations:** 1 Department of Precision Instrument, Tsinghua University, Beijng 100084, China; yang-ht17@mails.tsinghua.edu.cn (H.Y.); zhoub@mail.tsinghua.edu.cn (B.Z.); xhf15@mails.tsinghua.edu.cn (H.X.); 2 Xi’an Research Institute of High Technology, Xi’an 710025, China; 15191918796@163.com

**Keywords:** thermal calibration, error compensation, tri-axial MEMS gyroscope, Lagrange interpolation

## Abstract

The micro-electro-mechanical inertial measurement unit (MEMS-IMU) has gradually become a research hotspot in the field of mid-low navigation, because of its advantages of low cost, small size, light weight, and low power consumption (CSWap). However, the performance of MEMS-IMUs can be severely degraded when subjected to temperature changes, especially gyroscopes. In order to make full use of the navigation accuracy, this paper proposes an optimized error calibration method for a tri-axial MEMS gyroscope across a full temperature range. First of all, a calibration error model is established which includes package misalignment error, sensor-to-sensor non-orthogonality error, scale factor, and bias. Then, a simple three-position positive/reversed test is undertaken by carrying out a single-axis temperature-controlled turntable at different reference temperature points. Lastly, the error compensation vector is obtained using the least squares method to establish an error matrix. It is worth mentioning that the error compensation vector at a known temperature point can be calculated through Lagrange interpolation; then, the outputs of the tri-axial MEMS gyroscope can be well compensated, eliminating the need for a recalibration step. The experimental results confirm the effectiveness of the proposed method, which is feasible and operational in engineering applications, and has a certain reference value.

## 1. Introduction

With the rapid development of micro-electro-mechanical systems (MEMS), they have become widely used in the fields of drones, smart phones, motion tracking systems, and health testing devices [1]. MEMS inertial devices are also moving toward being low-cost, lightweight, high-precision devices, with a low power consumption and a small size. As one of the core inertial devices, the MEMS gyroscope has a relatively low accuracy due to differences in principles, manufacturing process, and environmental influences, whose errors mainly include random error and deterministic error. The random error generally adopts the Allan variance method to identify its error coefficients. The deterministic error includes the bias, nonlinear error, non-orthogonality error, temperature drift, and so forth. It is possible to eliminate most of the errors and improve the measurement accuracy by establishing an accurate mathematical error model and optimizing the compensation calibration. In Delgado’s research [2], a simple procedure for calibrating gyroscopes was proposed by a direct reversal of the coefficient matrix to estimate the sensor parameters through the automatic camera pan base. Zeng et al. [3] used a shaking table to calibrate the error of the gyroscope through the average method, the harmonic analysis method, and the least squares method, and explained the conditions and the estimation accuracy of each method. Golovan et al. [4] analyzed the observability of the potential estimation problem and proposed a calibration method for IMUs (inertial measurement units) on a two-axis turntable. This method is simple and all error coefficients can be estimated. In Wang’s research [5], the self-calibration of each error coefficient of the inertial device was performed by using the double-loop extended Kalman filter. The MEMS inertial device was calibrated for package errors using the direction of the vector cross product of the two-axis MEMS accelerometers in the same plane in the work by Li [6]. Eminoglu et al. [7] adopted a background calibration method to eliminate the scale factor error and bias by measuring the ratio of the oscillation frequency variation and calibration signal in the driving channel. In Prikhodko’s research [8], the driving force was applied by periodically reversing the polarity of the resonator to improve the bias instability of the low-cost MEMS vibratory gyroscope. In the work by Shen [9], a combination of linear calibration and wavelet signal processing was utilized to improve the accuracy and performance of the MEMS inertial sensor modules. In research by Jia [10], a complete error model and compensation scheme was established, which eliminated some of the error coefficients of the MEMS-IMU by the rotation modulation technique, and proved the feasibility of the method through static and dynamic tests. Further, different optimization schemes were excited in the error coefficients by rotating multiple positions [11,12,13,14]. At the same time, from the perspective of optimizing the identification algorithm, scholars have proposed various constructive ideas. In Li’s work [15], fuzzy logic was used to model and compensate for the error by analyzing the nonlinearity and asymmetry of the MEMS gyroscope scale factor. Secer et al. [16] established a deterministic error model and used a particle swarm optimization algorithm to identify the gyroscope error coefficients. Tedaldi et al. [17] assumed that the multi-position rotation scheme was used under the premise of a stable temperature and gravitational field, and the static filter was used to calibrate the error coefficient of a three-axis gyroscope. In the work by Kim [18], the error mathematical model of the MEMS-IMU was established, and the calibration test was carried out on a two axis high-accuracy rotary table, through nonlinear Gauss-Newton regression logic theory. In Lee’s research [19], the Fourier transform method was adopted to calibrate the bias, the scale factor, and the package misalignment error coefficient of the MEMS gyroscope, which achieved a certain improvement in the calculation time. In Eldiasty’s paper [20], the noise characteristics of MEMS inertial devices were tested using the Allan variance and least squares spectral analysis, respectively, and it was concluded that the static characteristics of the noise mainly depend on the cut-off frequency. In the research by Aggarwal [21], the six-position flipping method was applied to calibrate the deterministic error coefficients. Abdel- Sasani et al. [22] pointed out that the true value of the bias and the scale factor will be different due to the temperature change during the operation and calibration processes. In fact, temperature mainly affects the parameters of the MEMS gyroscope material properties, circuit parameters, air viscosity, capacitance, and dielectric constant, which indirectly affect the output of the MEMS gyroscope. Therefore, an accurate error modeling calibration method for temperature is essential.

To the best of our knowledge, the error calibration method at a single temperature is relatively mature. However, it is worth noting that the scale factor and bias of the tri-axial MEMS gyroscope change with the temperature [20,23]. The error compensation vector is different at different temperatures. Therefore, it is necessary to calibrate the error coefficient over a full temperature range to improve the accuracy and stability of tri-axial MEMS gyroscopes. Based on the above analysis, compared to existing error calibration methods such as multi-axis turntables and different optimization algorithms, this paper proposes a simple and practical error calibration method for a tri-axial MEMS gyroscope using a single-axis temperature-controlled turntable at different temperatures. Firstly, a flexible three-position positive/reversed test was carried out, and the least squares method was adopted to calculate the error coefficients, which includes misalignment errors, the scale factor, and bias at reference temperature points. Then, Lagrange interpolation was skillfully applied to fit the error compensation vector within the usable temperature range. Afterwards, the trend of the error coefficients with the temperature was acquired. The experimental results confirmed the validity and superiority of the proposed method. It is worth mentioning that as long as the temperature value can be obtained in the actual application process, the error coefficients of the known temperature points can be obtained and the error can be directly compensated for, which eliminates the cumbersome steps of recalibration.

This paper is organized as follows: Section 2 explains the basic principles of the Lagrange interpolation method. Section 3 describes the tri-axial MEMS gyroscope error model, including package misalignment error, sensor-to-sensor non-orthogonality error, scale factor, and bias. Then, an optimized calibration method and procedure for error calibration for the full temperature range is introduced to improve the accuracy and stability of the tri-axial MEMS gyroscope. Section 4 covers experiments using a single-axis temperature-controlled turntable and analyzes the calibration results, demonstrating the validity of the proposed method. A discussion and conclusions are given in Section 5.

## 2. Lagrange Interpolation

In numerical analysis, Lagrange interpolation is a polynomial function that is solved by knowing several points [24]. It is a unique algebraic interpolation which is easy to program and understand. The numerical calculation is stable and continuous in the defined interval. Lagrange interpolation has a wide range of applications in mathematics, physics, computers, and other fields. In the research by Mofdi [25], a new semi-Lagrange method was introduced to employ the finite element method on triangular meshes for spatial discretization, which was based on combining the modified method of characteristics with a high-order interpolating procedure. In Shi’s work [26], different strategies were compared to extract the energy spectra from a velocity field defined on a scattered set of points, so as to improve the turbulence modeling in a Lagrange framework. In Labanda’s research [27], a path-following strategy was proposed to localize cohesive cracks based on an energy release criterion model by implementing an augmented Lagrange formulation. In the work by Zhou [28], the Lagrange interpolation method was introduced and combined with the weighted average Lagrange interpolation model. In He’s research [29], it was used for a detailed analysis and discussion on obtaining GPS (Global Positioning System) satellites with higher and higher sampling rates of orbital locations. In the research by Ye [30], a novel adaptive image scaling algorithm with third-order Lagrange interpolation was proposed to obtain better quality real-time image scaling.

The basic principle of Lagrange interpolation can be expressed as:

Assuming a given k+1 value point, $\left(x_{0},y_{0}\right),\ldots ,\left(x_{k},y_{k}\right)$, $x_{j}$ corresponds to the position of the argument and $y_{j}$ corresponds to the value of the function at this position.

For any different $x_{j}$, if the values of $y_{j}$ is different from each other, Lagrange interpolation polynomials can be obtained:

(1) $$L(x)=\sum_{j=0}^{k}y_{j}l_{j}(x)\;$$

where each $l_{j}(x)$ is an interpolation basis function expressed as follows:

(2) $$l(x)=\prod_{i=0,i\neq j}^{k}\frac{x-x_{i}}{x_{j}-x_{i}}=\frac{x-x_{0}}{x_{j}-x_{0}}\ldots \frac{x-x_{j-1}}{x_{j}-x_{j-1}}\ldots \frac{x-x_{k}}{x_{j}-x_{k}}$$

## 3. Calibration Method

Calibration is the process of determining the coefficient of the output consistent with the reference information, which is achieved by comparing the known input with the output of the inertial device [31]. The calibration process includes two aspects: the calibration scheme and the calibration algorithm. This section first describes the error model that includes the package misalignment error, sensor-to-sensor non-orthogonality error, scale factor, and bias. For low-precision IMUs, because the earth rotation rate is much smaller than its bias output, it is necessary to use a turntable to calibrate the error coefficients at different positions. Then, a simple and easy three-position forward/backward calibration scheme was designed. The least squares method was used to calibrate the error of the tri-axial MEMS gyroscope, which is the most commonly used method in error parameters estimation [32,33,34,35,36,37].

### 3.1. Error Model

The tri-axial MEMS gyroscope error model includes bias, scale factor, random error (which is related to temperature), the non-orthogonality error between the tri-axial MEMS gyroscope axes, and the package misalignment error between the true package of the sensitive axes and body axes. According to the rigid body rotation theory [38], since the navigation coordinate system and the carrier coordinate system are both rectangular coordinate systems, and the axes are maintained at right angles, the coordinate system can be understood as a rigid body. When only the angular positional relationship between two coordinate systems is studied, the origin of a coordinate system is coincident with the origin of another coordinate system, which can be determined by translation. Therefore, the spatial angular position relationship between the two coordinate systems can be understood as the fixed point rotation of the rigid body. The package misalignment error can be defined as that which occurs when three rotation angles are sequentially rotated in the order of Z→X→Y to obtain the rotation matrix.

(3) $$C_{1}=\left[\begin{array}{ccc} \cos\theta_{z} & -\sin\theta_{z} & 0\\ \sin\theta_{z} & \cos\theta_{z} & 0\\ 0 & 0 & 1 \end{array}\right]$$

(4) $$C_{2}=\left[\begin{array}{ccc} 1 & 0 & 0\\ 0 & \cos\theta_{x} & \sin\theta_{x}\\ 0 & -\sin\theta_{x} & \cos\theta_{x} \end{array}\right]\;$$

(5) $$C_{3}=\left[\begin{array}{ccc} \cos\theta_{y} & 0 & -\sin\theta_{y}\\ 0 & 1 & 0\\ \sin\theta_{y} & 0 & \cos\theta_{y} \end{array}\right]$$

When $\theta_{x},\theta_{y},\theta_{z}$ are small angles, which can be ignored as high-order quantities between small angles, then the package misalignment error matrix can be approximated as:

(6) $$C_{b}^{n}=C_{3}C_{2}C_{1}\approx \left[\begin{array}{ccc} 1 & -\theta_{z} & \theta_{y}\\ \theta_{z} & 1 & -\theta_{x}\\ -\theta_{y} & \theta_{x} & 1 \end{array}\right]\;$$

The non-orthogonality error between the tri-axial MEMS gyroscope can also be similarly defined as:

(7) $$M\approx \left[\begin{array}{ccc} 1 & \beta_{yx} & \beta_{zx}\\ \beta_{xy} & 1 & \beta_{zy}\\ \beta_{xz} & \beta_{yz} & 1 \end{array}\right]\;$$

Here, $\beta_{ji},\left(i,j=x,\;y,\;z,\;i\neq j\right)$ represents the non-orthogonality error of i with respect to j.

The tri-axial MEMS gyroscope error model can be acquired:

(8) $$\left[\begin{array}{c} w_{x}\\ w_{y}\\ w_{z} \end{array}\right]=K_{T^{\prime }}C_{b}^{n}M\left[\begin{array}{c} {\tilde{w}}_{x}\\ {\tilde{w}}_{y}\\ {\tilde{w}}_{z} \end{array}\right]+\left[\begin{array}{c} B_{T^{\prime }x}\\ B_{T^{\prime }y}\\ B_{T^{\prime }z} \end{array}\right]+\left[\begin{array}{c} v_{x}\\ v_{y}\\ v_{z} \end{array}\right]\;$$

where $v_{i}$ (i=x, y, z, the same below) represents the measurement error of the MEMS gyroscope output, which can be expressed as Gaussian white noise. $w,\tilde{w}$ denotes the gyroscope output and the reference input value, respectively. $K_{T^{\prime }}$ and $B_{T^{\prime }i}$ indicate that the scale factor and bias vary with temperature. Since the interpolation method is used in this paper to calculate the values at different temperatures, it is not necessary to obtain definite expressions concerning temperature, the temperature gradient, and temperature change rate. Instead, they can be defined as:

(9) $$K_{T^{\prime }}=f\left(\lambda_{j},\;T,\;\Delta T,\;\frac{dT}{dt}\right)\;$$

(10) $$B_{T^{\prime }i}=f\left(\xi_{j},\;T,\;\Delta T,\;\frac{dT}{dt}\right)\;$$

where $\lambda_{j},\xi_{j}$ represents the corresponding coefficient value.

Let:

(11) $$K=K_{T^{\prime }}C_{b}^{n}M=\left[\begin{array}{ccc} K_{xx} & K_{yx} & K_{zx}\\ K_{xy} & K_{yy} & K_{zy}\\ K_{xz} & K_{yz} & K_{zz} \end{array}\right]\;$$

The error model of the tri-axial MEMS gyroscope can be summarized as:

(12) $$\left[\begin{array}{c} w_{x}\\ w_{y}\\ w_{z} \end{array}\right]=K\left[\begin{array}{c} {\tilde{w}}_{x}\\ {\tilde{w}}_{y}\\ {\tilde{w}}_{z} \end{array}\right]+\left[\begin{array}{c} B_{T^{\prime }x}\\ B_{T^{\prime }y}\\ B_{T^{\prime }z} \end{array}\right]+\left[\begin{array}{c} v_{x}\\ v_{y}\\ v_{z} \end{array}\right]\;$$

### 3.2. Calibration Scheme

**Figure 1:**
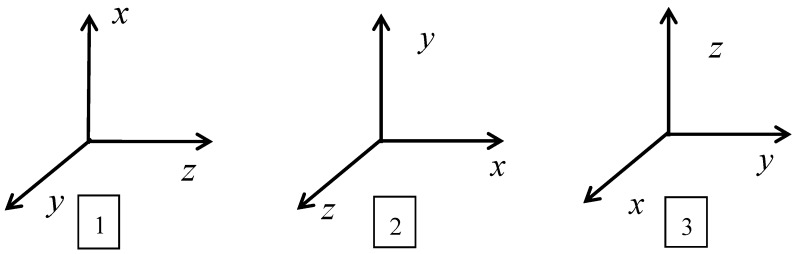
Three-position forward-reverse calibration diagram.

In the calibration process, the package misalignment error and scale factor are observable only when the MEMS-IMU is rotating. Therefore, it is necessary to provide as much angular rate input as possible in each axis direction to excite the error parameters. This paper adopts a single-axis temperature-controlled turntable to identify error by performing three-position forward-reverse tests at different temperatures, as shown in Figure 1.

Each rotation speed point is rotated in turn to collect the data, with a stable temperature and rotation speed in Table 1 and Table 2 respectively, through different locations and different temperature points.

After holding at each reference temperature point for 1 h, data at each rate point for 1 min is collected for calibration. Equation (12) can be written in the following form:

(13) $$\left[\begin{array}{c} w_{x}\\ w_{y}\\ w_{z} \end{array}\right]=K_{g}\Omega \;$$

where:

(14) $$K_{g}=[\begin{array}{cccccccccccc} K_{xx} & K_{yy} & K_{zz} & K_{yx} & K_{zx} & K_{xy} & K_{zy} & K_{xz} & K_{yz} & B_{x} & B_{y} & B_{z} \end{array}]\;$$

(15) $$\Omega ={\left[\begin{array}{cccccccccccc} {\tilde{w}}_{x} & 0 & 0 & {\tilde{w}}_{y} & {\tilde{w}}_{z} & 0 & 0 & 0 & 0 & 1 & 0 & 0\\ 0 & {\tilde{w}}_{y} & 0 & 0 & 0 & {\tilde{w}}_{x} & {\tilde{w}}_{z} & 0 & 0 & 0 & 1 & 0\\ 0 & 0 & {\tilde{w}}_{z} & 0 & 0 & 0 & 0 & {\tilde{w}}_{x} & {\tilde{w}}_{y} & 0 & 0 & 1 \end{array}\right]}^{T}$$

where $B_{x},B_{y},B_{z}$ is the bias of the tri-axial MEMS gyroscope, and ${\tilde{w}}_{x},{\tilde{w}}_{y},{\tilde{w}}_{z}$ is the angular velocity reference input.

At the time of temperature $T_{i}$, the matrix of each reference input point is:

(16) $$\tilde{\Omega }=\left[\begin{array}{c} {\hat{\Omega }}_{x}\\ {\hat{\Omega }}_{y}\\ {\hat{\Omega }}_{z} \end{array}\right]\;$$

where i=x,y, z.

(17) $${\hat{\Omega }}_{i}={\left[\begin{array}{cccccccccccc} {\tilde{w}}_{xi1} & 0 & 0 & {\tilde{w}}_{yi1} & {\tilde{w}}_{zi1} & 0 & 0 & 0 & 0 & 1 & 0 & 0\\ 0 & {\tilde{w}}_{yi1} & 0 & 0 & 0 & {\tilde{w}}_{xi1} & {\tilde{w}}_{zi1} & 0 & 0 & 0 & 1 & 0\\ 0 & 0 & {\tilde{w}}_{zi1} & 0 & 0 & 0 & 0 & {\tilde{w}}_{xi1} & {\tilde{w}}_{yi1} & 0 & 0 & 1\\ \vdots & \vdots & \vdots & \vdots & \vdots & \vdots & \vdots & \vdots & \vdots & \vdots & \vdots & \vdots \\ {\tilde{w}}_{xi23} & 0 & 0 & {\tilde{w}}_{yi23} & {\tilde{w}}_{zi23} & 0 & 0 & 0 & 0 & 1 & 0 & 0\\ 0 & {\tilde{w}}_{yi23} & 0 & 0 & 0 & {\tilde{w}}_{xi23} & {\tilde{w}}_{zi23} & 0 & 0 & 0 & 1 & 0\\ 0 & 0 & {\tilde{w}}_{zi23} & 0 & 0 & 0 & 0 & {\tilde{w}}_{xi23} & {\tilde{w}}_{yi23} & 0 & 0 & 1 \end{array}\right]}_{69\times 12}^{T}$$

(18) $$\hat{w}=\left[\begin{array}{ccc} {\bar{w}}_{x} & {\bar{w}}_{y} & {\bar{w}}_{z} \end{array}\right]\;$$

where ${\bar{w}}_{i}={\left[\begin{array}{ccccccc} {\bar{w}}_{xi1} & {\bar{w}}_{yi1} & {\bar{w}}_{zi1} & \cdots & {\bar{w}}_{xi23} & {\bar{w}}_{yi23} & {\bar{w}}_{zi23} \end{array}\right]}_{1\times 69}$.

In addition, the error coefficient $K_{gT_{i}}$ at the time of temperature $T_{i}$ can be obtained by least squares fitting:

(19) $$K_{gT_{i}}=\hat{w}\cdot {\hat{\Omega }}^{T}\cdot {\left(\hat{\Omega }\cdot {\hat{\Omega }}^{T}\right)}^{-1}\;$$

In order to obtain the error vector matrix of 10 temperature points, the error coefficients of different temperature points are calculated in turn:

(20) $${\tilde{K}}_{gT}=\left[\begin{array}{cccc} K_{gT_{1}} & K_{gT_{2}} & \ldots \ldots & K_{gT_{10}} \end{array}\right]\;$$

When the temperature $T_{k}$ is known, the error coefficient vectors at the temperature points $T_{i}{}_{-2},T_{i}{}_{-1},T_{i}$ are $K_{gT_{\left(i-2\right)}},K_{gT_{\left(i-1\right)}},K_{gT_{\left(i\right)}}$, respectively. According to the Lagrange interpolation method, the error coefficient vector at temperature $T_{k}\left(T_{i-1}<T_{k}<T_{i}\right)$ can be obtained:

(21) $$K_{gT_{k}}=K_{gT_{\left(i-2\right)}}L_{2}+K_{gT_{\left(i-1\right)}}L_{1}+K_{gT_{\left(i\right)}}L_{0}\;$$

where:

(22) $$\{\begin{array}{c} L_{0}=\frac{\left(T_{k}-T_{i-2}\right)\cdot \left(T_{k}-T_{i-1}\right)}{\left(T_{i}-T_{i-2}\right)\cdot \left(T_{i}-T_{i-1}\right)}\\ L_{1}=\frac{\left(T_{k}-T_{i-2}\right)\cdot \left(T_{k}-T_{i}\right)}{\left(T_{i-1}-T_{i-2}\right)\cdot \left(T_{i-1}-T_{i}\right)}\\ L_{2}=\frac{\left(T_{k}-T_{i}\right)\cdot \left(T_{k}-T_{i-1}\right)}{\left(T_{i-2}-T_{i}\right)\cdot \left(T_{i-2}-T_{i-1}\right)} \end{array}\;$$

**Figure 2:**
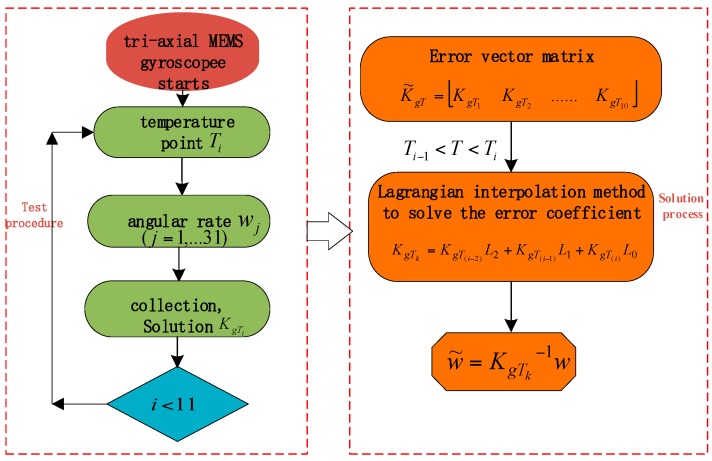
Error compensation flow chart.

In this way, the error vector at time $T_{i}$ can be obtained and the output of the tri-axial MEMS gyroscope can be compensated effectively. The specific flow chart is presented in Figure 2.

## 4. Test Results and Analysis

**Figure 3:**
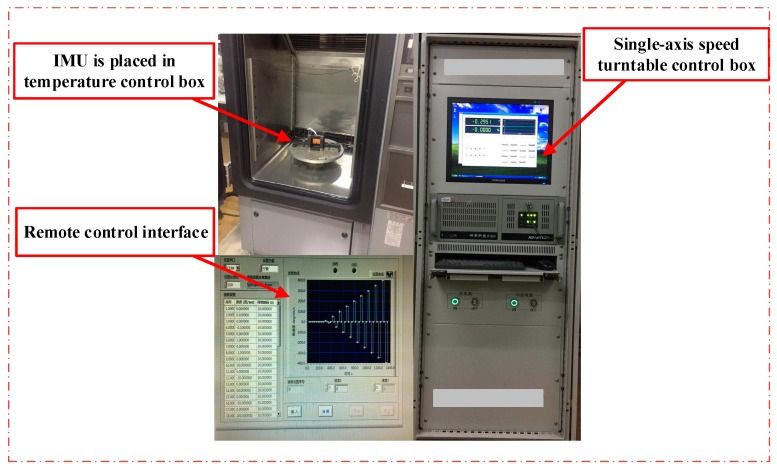
Block diagram of the test device.

A single axis temperature-controlled turntable provides accurate angular velocity and temperature input in Figure 3. Based on the above tri-axial MEMS gyroscope error model and calibration method, the MEMS-IMU was fixed on a high-precision hexahedron, and the experiments were performed at different temperature points to acquire the error vector matrix, after which the calculation results were compared and analyzed.

**Figure 4:**
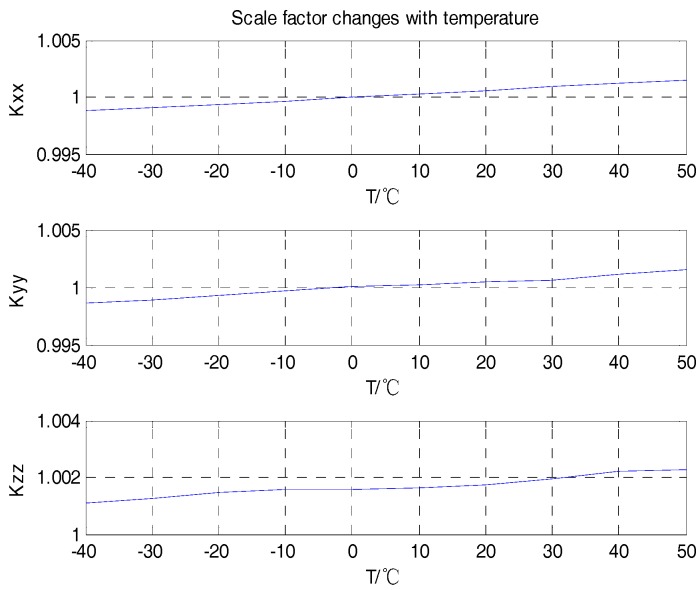
Scale factor varies with temperature.

**Figure 5:**
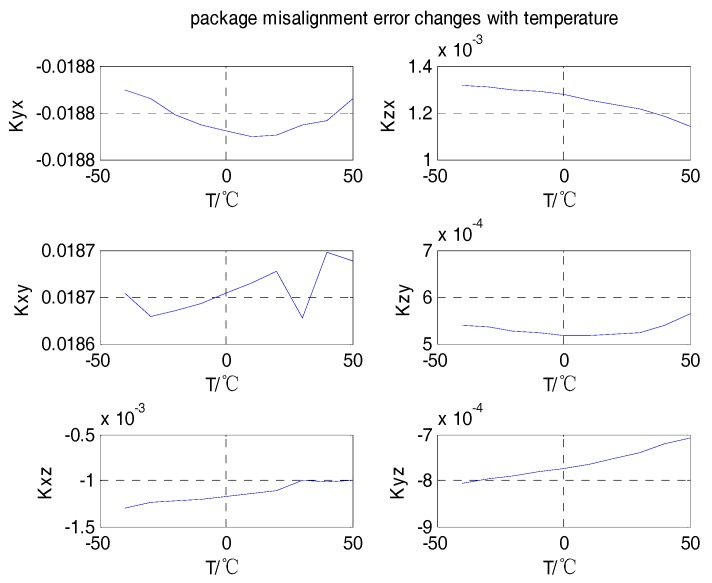
Package misalignment error varies with temperature.

**Figure 6:**
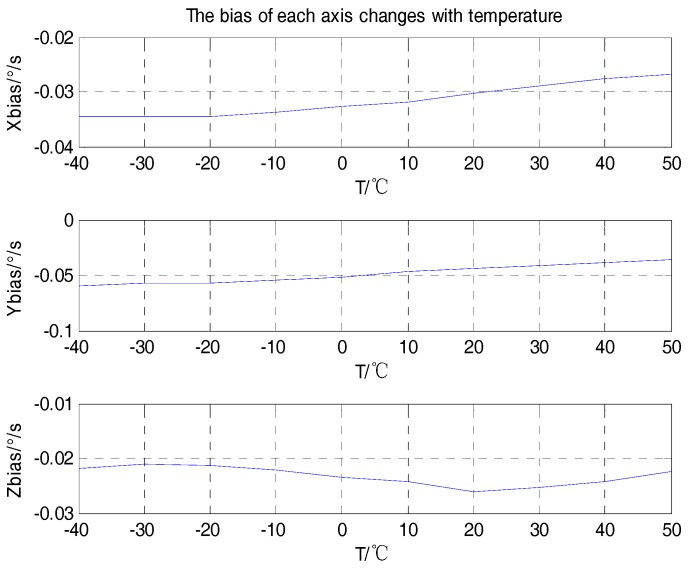
Bias varies with temperature.

Firstly, the curves of the scale factor, package misalignment error, and bias with temperature were analyzed, as shown in Figure 4, Figure 5 and Figure 6.

From the analyses shown in Figure 4, Figure 5 and Figure 6, we can see that the scale factors $K_{xx},\;K_{yy},\;K_{zz}$ of the tri-axial MEMS gyroscope showed a linear relationship with temperature. The bias output $X_{bias},\;Y_{bias},\;Z_{bias}^{}$ also changed with temperature. The values of the package misalignment error $K_{xy},\;K_{yx}$ were large, which was identified as the main cause of package misalignment error, but unexpected non-orthogonality error was generated when there was a high dynamic environment. The other package misalignment errors $K_{xz},\;K_{zx},\;K_{yz},\;K_{zy}$ were relatively small. Furthermore, as can be seen from the trend, the change of temperature did not affect the value of the package misalignment error.

**Figure 7:**
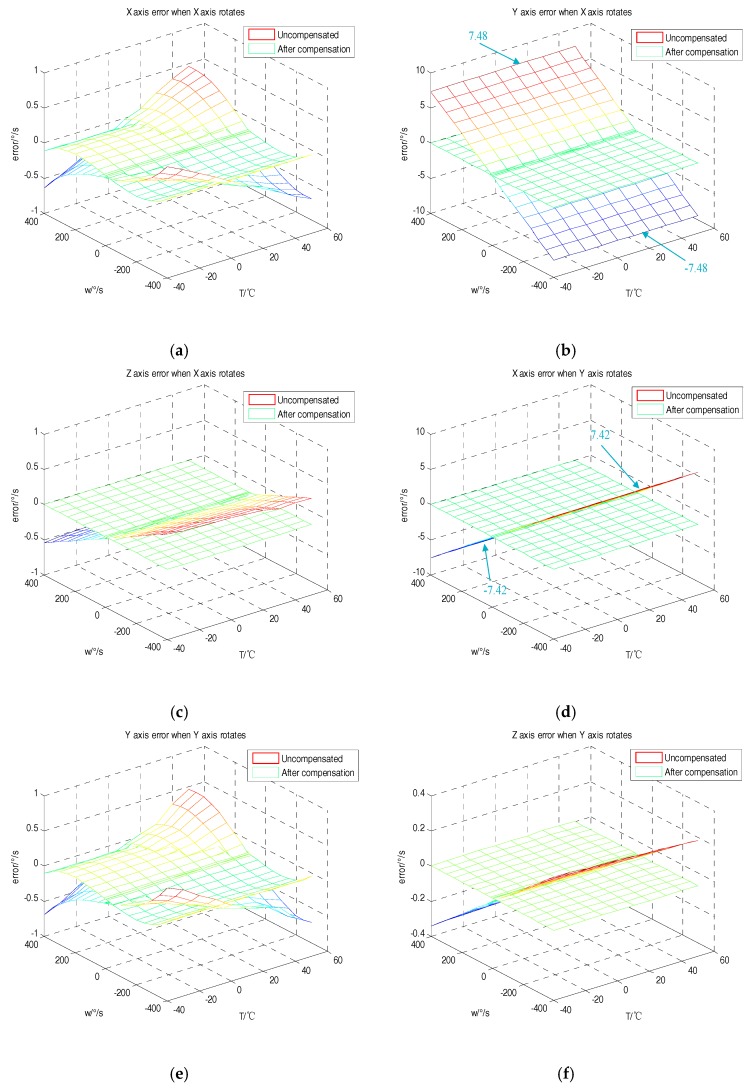

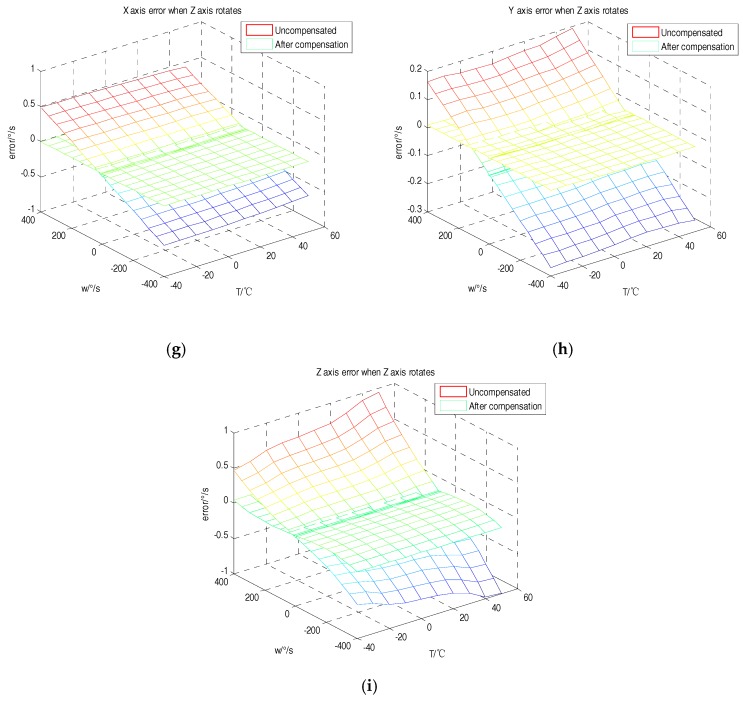
Error distribution before and after compensation. (**a**) Error distribution of the X-axis when the X-axis rotates; (**b**) error distribution of the Y-axis when the X-axis rotates; (**c**) error distribution of the Z-axis when the X-axis rotates; (**d**) error distribution of the X-axis when the Y-axis rotates; (**e**) error distribution of the Y-axis when the Y-axis rotates; (**f**) error distribution of the Z-axis when the Y-axis rotates; (**g**) error distribution of the X-axis when the Z-axis rotates; (**h**) error distribution of the Y-axis when the Z-axis rotates; (**i**) error distribution of the Z-axis when the Z-axis rotates.

The three-dimensional figure of each rotation speed and temperature was shown by plotting the error of each axis of the gyroscope before and after compensation, as in Figure 7. According to the analysis, the tri-axial MEMS gyroscope was compensated using the error coefficients at different temperatures. It can be clearly seen that the error before and after the compensation was significantly reduced. From the analysis of Figure 7b,d, in the range of −400 °/s to 400 °/s, the non-orthogonality error of the Y-axis when the X-axis rotates and the non-orthogonality error of the X-axis when the Y-axis rotates were 7.48 °/s–−7.48 °/s and −7.42 °/s–7.42 °/s, respectively. This result is consistent with the fluctuations in the package misalignment error between the two axes analyzed earlier, which were around −0.0188 and 0.0187. Since the package misalignment errors between the Z-axis and the X-, Y-axes were small, the non-orthogonality error value had a smaller fluctuation range. However, the proposed method can also compensate well for the non-orthogonality error when the Z-axis rotates. This proves the correctness and feasibility of the proposed method.

Mean and Root Mean Square (RMS) error is used as a direct comparison here. From the data analysis in Table 3, the proposed method has a significant reduction in both Mean and RMS compared to the uncompensated method.

Subsequently, we further compared the compensation effect of the proposed method with the traditional method. The traditional method refers to fitting a set of error calibration vectors by the least squares method under a single temperature condition. However, when the temperature changes, the components of the three-axial MEMS gyroscope suffer from distortion or stress, so the nonlinear scale factor or bias also changes. Modeling these errors with polynomials in a single model does not realize an accurate evaluation. After calibration, there are still some residuals in the angular rate information. This was proven through the following test. The values of the X-, Y-, and Z-axes rotating at full speed were acquired at 25 °C. The error vector at this temperature was fitted using the proposed method:

(23) $$\begin{array}{l} K_{g}=[\begin{array}{cccccc} 1.0005 & 1.0005 & 1.0017 & -0.0188 & 0.0012 & 0.0187 \end{array}\\ \begin{array}{cccccc} 5.2027\;\times \;10^{-4} & -0.0011 & -7.5133\;\times \;10^{-4} & -0.0303 & -0.0435 & -0.0261 \end{array}] \end{array}$$

The error vector solved by the traditional method is:

(24) $$\begin{array}{c} K_{g}{}^{\prime }=[\begin{array}{cccccc} 1.001 & 1.0001 & 1.0017 & -0.018 & 0.0013 & 0.0187 \end{array}\\ \begin{array}{cccccc} 5.3089\;\times \;10^{-4} & -0.0011 & -7.6320\;\times \;10^{-4} & -0.0316 & -0.0488 & -0.0232 \end{array}] \end{array}$$

**Figure 8:**
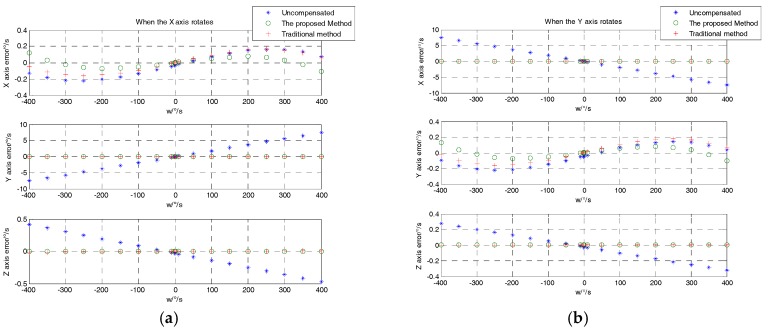

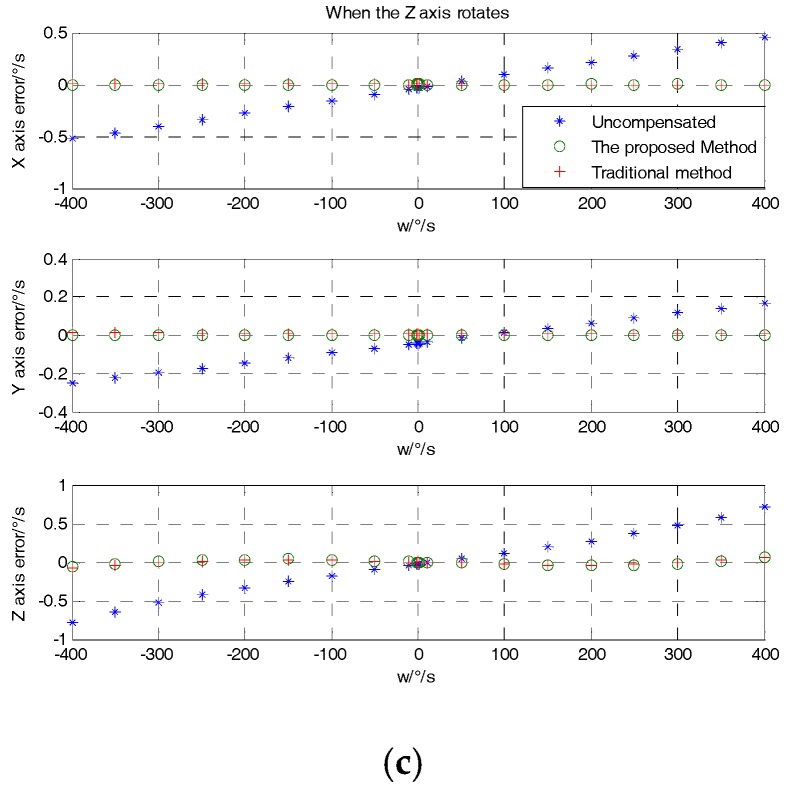
Error distribution of each axis (determined using the uncompensated, proposed, and traditional methods). (**a**) Error distribution when the X-axis rotates; (**b**) error distribution when the Y-axis rotates; (**c**) error distribution when the Z-axis rotates.

**Figure 9:**
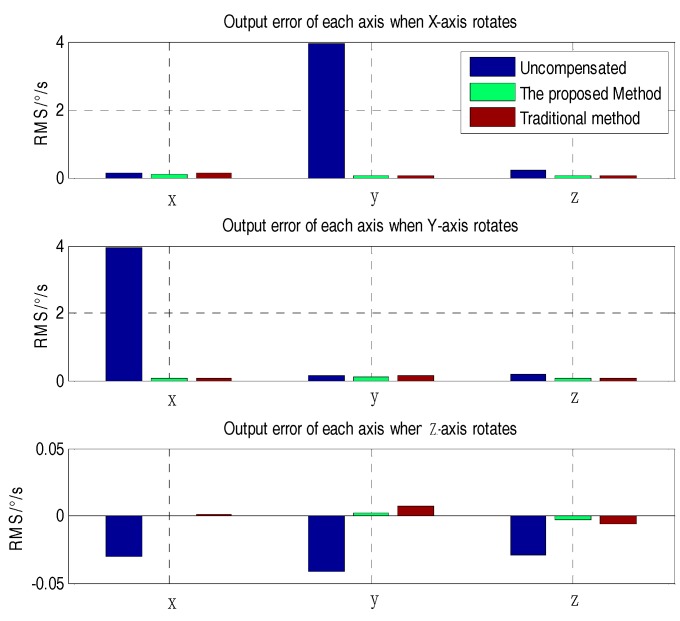
RMS of each axis when each axis rotates (determined using the uncompensated, proposed, and traditional methods).

From Figure 8, it can be seen that the proposed method and the traditional method have better compensation results than the uncompensated one. To further analyze the effect of the two methods, the (RMS) error of each axis was adopted to characterize the compensation effect. From Figure 9, it can be seen that the RMS error of the proposed method and the traditional method were obviously lower than that of the uncompensated condition. In contrast with traditional method and the proposed method, the X-axis error when the X-axis rotates and the Y-axis error when the Y-axis rotates were decreased by 26.75% and 25.64%, respectively. The Y- and Z-axis error compensation effects when the X-axis rotates were slightly improved. The X- and Z-axis error compensation effect when the Y-axis rotates was basically the same. Due to the large package misalignment error between the X- and Y-axes, when rotating within ±400°/s, the non-orthogonality error compensation effect reached an approximate level. However, when the Z-axis rotates, the X, Y, and Z errors were significantly reduced by 55.50%, 75.36% and 48.33%, respectively, as compared to the conventional methods. Specific comparisons are shown in Table 4.

## 5. Discussion and Conclusions

Error calibration is crucial to improve the accuracy and stability of MEMS inertial devices. This paper presented an optimized tri-axial MEMS gyroscope error calibration method. First, an error model was established including the package misalignment error, non-orthogonality error, scale factor, and bias. Then, the tri-axial MEMS gyroscope was calibrated at different temperature reference points by a simple three-position positive/reversed method. The strategy applied in this paper was that when there was a known temperature, the Lagrange interpolation method was used to fit the current temperature error matrix to compensate for the tri-axial MEMS gyroscope output. Through experimental analysis, it was found that when the misalignment error is large, the accuracy of the compensation is comparable to that of the traditional method. When the misalignment error is small, the X-, Y-, and Z-axis errors decrease by 55.50%, 75.36%, and 48.33%, respectively. Therefore, reducing the occurrence of errors as much as possible in the process of manufacturing and installation, so as to increase the error compensation accuracy, is required. At the same time, the method proposed in this paper proposes a new idea for the practical application of tri-axial MEMS gyroscope error calibration: only one calibration experiment under multiple temperature points is needed to establish an error vector matrix, since as long as the temperature value can be obtained during the compensation process, the output can be compensated. This has a certain engineering application value. Further investigations into the calibration algorithm in all error coefficients is required, such as in nonlinear error and g-sensitive error. Finally, the next important task is to embed the calibration matrix into the control program for real-time calibration.

## Figures and Tables

**Table 1:** Calibration temperature point (°C).

Number	$T_{1}\;$	$T_{2}\;$	$T_{3}\;$	$T_{4}\;$	$T_{5}\;$	$T_{6}\;$	$T_{7}\;$	$T_{8}\;$	$T_{9}\;$	$T_{10}\;$
Value	−40	−30	−20	−10	0	10	20	30	40	50

**Table 2:** Calibration angular rate point (°/s).

0	±0.1	±1	±10	±50	±100	±150	±200	±250	±300	±400

**Table 3:** Comparison of the Mean and Root Mean Square (RMS) (uncompensated method and the proposed method).

			Uncompensated (°/s)	Proposed Method (°/s)
X-axis rotates	X-axis	Mean	−0.0302	0.0013
		RMS	0.1783	0.0532
	Y-axis	Mean	−0.0538	−0.0050
		RMS	4.0177	0.0011
	Z-axis	Mean	−0.0242	−9.9029 × 10^−3^
		RMS	0.2459	0.0015
Y-axis rotates	X-axis	Mean	−0.0330	−0.0014
		RMS	4.0446	0.0018
	Y-axis	Mean	−0.0471	0.0017
		RMS	0.1789	0.0568
	Z-axis	Mean	−0.0197	0.0035
		RMS	0.1644	0.0011
Z-axis rotates	X-axis	Mean	−0.0315	1.260 × 10^−4^
		RMS	0.2695	9.253 × 10^−4^
	Y-axis	Mean	−0.0455	0.0033
		RMS	0.1143	0.0016
	Z-axis	Mean	−0.0257	−0.0025
		RMS	0.3621	0.0263

**Table 4:** RMS error difference comparison table.

		Uncompensated Method (°/s)	Traditional Method (°/s)	The Proposed Method (°/s)	Compared to Uncompensated Method (%)	Compared to Traditional Method (%)
X-axis rotates	X-axis	0.1453	0.1271	0.0931	35.93	26.75
	Y-axis	3.9339	0.0474	0.0473	98.78	2.110
	Z-axis	0.2391	0.0492	0.0488	79.59	8.130
Y-axis rotates	X-axis	3.9576	0.0467	0.0467	98.82	0
	Y-axis	0.1374	0.1287	0.0957	30.35	25.64
	Z-axis	0.1656	0.0486	0.0486	70.65	0
Z-axis rotates	X-axis	−0.0307	9.8550 × 10^−4^	−4.3855 × 10^−4^	98.57	55.50
	Y-axis	−0.0418	0.0069	0.0017	95.93	75.36
	Z-axis	−0.0292	−0.0060	−0.0031	89.38	48.33
